# *Ficus erecta* Thunb. Leaves Ameliorate Cognitive Deficit and Neuronal Damage in a Mouse Model of Amyloid-β-Induced Alzheimer’s Disease

**DOI:** 10.3389/fphar.2021.607403

**Published:** 2021-04-15

**Authors:** Eunjin Sohn, Yu Jin Kim, Joo-Hwan Kim, Soo-Jin Jeong

**Affiliations:** ^1^ Clinical Medicine Division, Korea Institute of Oriental Medicine, Daejeon, South Korea; ^2^ Department of Life Science, Gachon University, Seongnam, South Korea

**Keywords:** amyloid-β, Alzheimer’s disease, *Ficus erecta Thunb.*, neuroinflammation, neuronal loss

## Abstract

Alzheimer’s disease (AD) pathogenesis is linked to amyloid plaque accumulation, neuronal loss, and brain inflammation. *Ficus erecta* Thunb. is a food and medicinal plant used to treat inflammatory diseases. Here, we investigated the neuroprotective effects of *F. erecta* Thunb. against cognitive deficit and neuronal damage in a mouse model of amyloid-β (Aβ)-induced AD. First, we confirmed the inhibitory effects of ethanol extracts of *F. erecta* (EEFE) leaves on Aβ aggregation *in vivo* and *in vitro*. Next, behavioral tests (passive avoidance task and Morris water maze test) revealed EEFE markedly improved cognitive impairment in Aβ-injected mice. Furthermore, EEFE reduced neuronal loss and the expression of neuronal nuclei (NeuN), a neuronal marker, in brain tissues of Aβ-injected mice. EEFE significantly reversed Aβ-induced suppression of cAMP response element-binding protein (CREB) phosphorylation and brain-derived neurotrophic factor (BDNF) expression, indicating neuroprotection was mediated by the CREB/BDNF signaling. Moreover, EEFE significantly suppressed the inflammatory cytokines interleukin 1beta (IL-1β) and tumor necrosis factor alpha (TNF-α), and expression of ionized calcium-binding adaptor molecule 1 (Iba-1), a marker of microglial activation, in brain tissues of Aβ-injected mice, suggesting anti-neuroinflammatory effects. Taken together, EEFE protects against cognitive deficit and neuronal damage in AD-like mice via activation of the CREB/BDNF signaling and upregulation of the inflammatory cytokines.

## Introduction

Alzheimer’s disease (AD) is a progressive and irreversible neurodegenerative disorder that leads to memory loss and learning deficit. The main trademark of AD pathology is amyloid-β (Aβ) peptide accumulation and the large aggregates (plaques) formation in the brain (Longo and Massa, 2004; Morroni et al., 2018). Therefore, prevention of Aβ aggregation and accumulation is a potential strategy for the treatment of AD. Although anti-Aβ treatment has failed in clinical trials, Aβ is still considered a key target molecule for developing novel AD therapies (Lim et al., 2019; Sikanyika et al., 2019). However, U.S. Food Drug Administration (FDA)-approved drugs for AD, namely tacrine, galantamine, rivastigmine, donepezil, and memantine, target acetylcholinesterase or the *N*-methyl-d-aspartate (NMDA) receptor, not Aβ; those drugs do not provide a cure and have low efficacy and severe side effects. In the face of these limitations, complementary and alternative therapies could offer a solution to block the progression of AD.

Many studies have reported the potential of natural products or medicinal plants to prevent or treat AD (Bui and Nguyen, 2017; Dey et al., 2017). For instance, *Ginkgo bilona* L. and its constituents ginkgolide and bilobalide have the clinical efficacy in mild to moderate AD patients compared with cholinesterase inhibitors (Mazza et al., 2006; Nasab et al., 2012). *Vitis amurnsis* Rupr extract and its active compound resveratrol have the neuroprotective effects againt Aβ-induced neurotoxicity *in vivo* and *in vitro* (Jang et al., 2007; Jeong et al., 2010). In addition, various medicinal plants and natural products were reported as neuroprotective agents such as *Curcuma longa* and curcumin (Hamaguchi et al., 2010), *Allium sativum* L. and s-allyl cysteine (Chauhan, 2006), and *Magnolia offinalis* and 4-o-methlyhonokiol (Lee et al., 2011) by exerting anti-oxidant, anti-amyloidogenic, and anti-inflammatory effects in an AD animal model, respectively. More recent studies have reported that aged garlic extract (AGE) enhances cognitive memory and reduces neuroinflammation in an Aβ-induced AD rat model (Nillert et al., 2017) and attenuates neuroinflammation in microglial cells (Qu et al., 2016). If proven to be effective and safe, these natural plants could be used for patients with AD (Habtemariam, 2019).

*Ficus erecta* Thunb. (hereinafter referred to simply as *F. erecta*) is a native plant distributed in seashore areas of Korea, Japan, and China. *F. erecta*, which is resistant to fungus, has been used as a foods and medicines for nephritis and arthritis, such as inflammatory diseases (Park et al., 2012; Yakushiji et al., 2012). Recent studies have reported that *F. erecta* leaves inhibit osteoporotic inflammatory factors (Yoon et al., 2007) and that *F. erecta* fruits exert anti-oxidant and thrombolytic activities (Abullah et al., 2018). Despite the traditional uses of *F. erecta*, scientific evidence of its pharmacological activities has been limited to the two above-mentioned articles. In the current study, we assessed the neuroprotective effects of *F. erecta* against cognitive deficit and neuronal damage in an Aβ-induced AD mouse model. Here, we report that EEFE has the potential to inhibit Aβ aggregation and protect against neuronal damage and inflammation via activation of the cAMP response element-binding protein (CREB)/brain-derived neurotrophic factor (BDNF) signaling and upregulation of the inflammatory cytokine levels in Aβ-injected (AD-like) mice.

## Materials and Methods

### Plant Extracts

The dried leaves and branches of *F. erecta* were kindly provided by the Korean Seed Association (Seongnam, Korea) and identified by Professor Joo-Hwan Kim (Gachon University, Seongnam, Korea). Voucher specimens (SCD-A-114-1 and SCD-A-114-2) were placed at the Clinical Medicine Division, Korea Institute of Oriental Medicine (Daejeon, Korea). The dried leaves (3.4 kg) and branches (4 kg) of *F. erecta* were extracted twice with aqueous ethanol using a vacuum extractor (COSMOS-660, Kyungseo Machine Co., Incheon, Korea) for 3 h at 80 ± 2°C. The filtered solution was concentrated using a vacuum evaporator and freeze-dried to obtain powdered leaf and branch extracts (636.13 and 293.31 g, respectively). The yield of EEFE was 18.71% (leaf extracts) and 7.33% (branch extracts).

### *In Vitro* Aβ_1–42_ Aggregation Assay

The Aβ_1–42_ aggregation assay was performed as described in our previous report (Lim et al., 2018) using the Thioflavin T β-Amyloid Aggregation kit (Cat. AS-72214, Anaspec, Inc., Fermont, CA, Unites States). The mixture of Aβ_1–42_ peptide solution and EEFE was combined with thioflavin T. Then, fluorescence signals were read at an excitation/emission wavelength = 440/484 nm at 37°C using a microplate reader (SpectraMax i3, Molecular Devices, Sunnyvale, CA, United States). Morin (Cat. M4008-2G, Sigma-Aldrich, St. Louis, MO, United States), positive control, was used as an Aβ aggregation inhibitor. Experiments were repeated three times and performed in triplicate. The inhibition (%) of Aβ_1–42_ aggregation was computed by following equation:

$$\text{Inhibition}\;\text{of}\;A\beta \;\text{aggregation}\left(\%\right)=\left(1-\frac{\text{Fluorescence}\;\text{of}\;A\beta -\text{treated}\;\text{sample}}{\text{Fluorescence}\;\text{of }\;\text{untreated}\;\text{sample}}\right)\;\times \;100.$$

### Animals and Intracerebroventricular Injection of Aβ Aggregates

Male C57BL6 mice weighing 24 ± 2 g were obtained from Orient Bio (Seoul, South Korea), and acclimated for 1 week prior to the study. Animals were given standard rodent diet (2918C; ENVIGO, Blackthorn, United Kingdom) and water *ad libitum*. They were maintained in individual acryl cages at a temperature of 23 ± 3°C, relative humidity of 55 ± 15%, ventilation frequency of 10–20 times/h, illumination intensity of 150–300 lux, and lights on from 8 AM until 8 PM (12:12 h light:dark cycle). The experiments were approved by the Institutional Animal Care and Use Committee of Nonclinical Research Institute, Chemon Inc. (IACUC Approval No. 18-M111) and was performed according to the National Institutes of Health (NIH) Guide for the Care and Use of Laboratory Animals.

**FIGURE 1:**
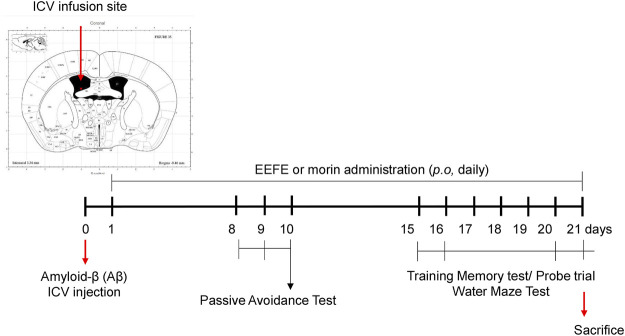
Timeline for EEFE administration and behavioral tests in the animal experiments. Amyloid-β aggregates were injected at intracerebroventricular (ICV) area with stereotaxic apparatus into male C57BL6 mice. EEFE (50 or 100 mg/kg) or morin (10 mg/kg) were orally treated daily for 20 days. The passive avoidance task was performed on the 8^th^–10^th^ day. Morris water maze test (MWM) was conducted on the 15^th^–21^st^ day. EEFE: ethanol extracts of *Ficus erecta*. Image of the ICV infusion site was made from Mouse Brain Atlas (labs.gaidi.ca). Atlas Source: Paxinos, George, and Keith B. J. Franklin. The mouse brain in stereotaxic coordinates: hard cover edition.

The Aβ_1–42_ peptide was obtained from Tocris Bioscience (Cat. 1428, Pittsburgh, PA, United States), dissolved to a concentration of 1 μg/μL in sterilized 0.1 M phosphate-buffered saline (PBS; pH 7.4), and incubated at −20°C for 4 weeks to encourage the formation of Aβ aggregates. Mice were anesthetized with mixed zoletil (tiletamine–zolazepam; Verbac, Carros, France) and rompun (Bayer, Leverkusen, Germany) (4:1 v/v) at dose of 1 ml/kg before Aβ injection. Intracerebroventricular (ICV) injection was performed as previously described (Lim et al., 2018; Lim et al., 2019; Franklin and Paxinos, 2008). The incubated Aβ aggregates were injected in the ICV area using a stereotaxic apparatus (Harvard Apparatus, Panlab, Sandiego, CA, United States) fixed at the following coordinates: anterior/posterior (AP) −0.5 mm, and mediolateral/lateral 1.0 mm dorsal/ventral 2.5 mm from Bregma. Aggregated Aβ_1–42_ (5 μL) was infused at a rate of 2 μL/min speeds. Saline was used as a control. At 24 h after the injection, experimental mice were assorted into five groups (*n* = 7/group): normal control mice (NOR), Aβ_1–42_-injected mice (Aβ), Aβ + EEFE-injected mice at 50 or 150 mg/kg/day (EEFE-50 or EEFE-150), and Aβ + morin-injected mice at 10 mg/kg/day (M-10). Animals received a nontoxic concentration of EEFE for 20 days. EEFE was dissolved in distilled water (vehicle). The experimental schedule, including Aβ injection, EEFE administration, and behavioral testing, is shown in Figure 1.

### Preparation of Brain Tissue Sections for Immunohistochemistry and Nissl Staining

At the end of experimental period, mice from each group were sacrificed under deep anesthesia. Three mouse brains from each group were perfused transcardially with saline and then fixed in 4% paraformaldehyde. Each of hippocampal and cortical tissues isolated from four mice of each group were immediately stored at −80°C until further analysis. Brain paraffin blocks were sliced into 4-μm-thick sections. The slides were deparaffinized and hydrated with xylene and sequentially of ethanol solution, respectively. Slides were quenched endogenous peroxidase activity with 0.3% hydrogen peroxide in methanol for 25 min and rinsed in PBS. For antigen retrieval, slides were boiled in citrate buffer (pH 6.0) in a microwave for 15 min. Slides were incubated with horse serum for blocking for 1 h at 37°C and then probed with primary anti-Aβ (AB_10988723, 1:250 dilution; Santa Cruz Biotechnology Inc., Dallas, TX, United States), anti-neuronal nuclei (NeuN) (AB_10711153, 1:250 dilution; Abcam, Cambridge, United Kingdom), and anti-ionized calcium-binding adaptor molecule 1 (Iba-1) (AB_839504, 1:100 dilution; Wako Pure Chemicals, Osaka, Japan) antibodies for overnight at 4°C. To detect Aβ, NeuN and Iba-1, the slides were incubated with labeled streptavidin–biotin for 30 min and visualized using diaminobenzidine tetrahydrochloride (DAB; Vector Laboratories, Burlingame, CA, United States). Slides were immersed in 1% cresyl violet acetate solution for Nissl staining, washed with water, and dehydrated with 90% and 100% ethanol for 5 min before mounting in xylene. Mounted slides were then captured at 400× magnification using a microscope (Olympus DP71, Tokyo, Japan). Image analysis was performed by blinded investigators using Image J software program (Java-based image processing program, NIH, Bethesda, MD, United States).

### Western Blot Analysis

Homogenized brain lysates (30 μg) were resolved on polyacrylamide gels and then transferred on 0.2-μm PVDF membranes using the Trans-Blot transfer system (Bio-Rad, Hercules, CA, United States). Blocked membranes with 5% nonfat dry milk were washed with Tris-buffered saline with 0.1% tween 20 (TBST) and then incubated with primary anti-BDNF (AB_10862052, 1:2000 dilution; Abcam), anti-phospho-CREB (AB_731734, 1:3000 dilution; Abcam), anti-total CREB (AB_2827810, Abcam), and anti-Aβ (AB_626669, 1:3000 dilution; Santa Cruz Biotechnology Inc.) antibodies for overnight at 4°C. Washed membranes with TBST were incubated with secondary antibodies anti-rabbit horseradish peroxidase (HRP). Immuno-reactive membranes were developed using the chemiluminescent substrate reagents (Cat. 34577, Amersham Bioscience, Piscataway, NJ, United States). Membranes were also incubated with anti-β-actin (AB_476743, 1:3000 dlution; Sigma-Aldrich, Saint Louis, MO, United States) for loading endogenous reference control. Protein bands were detected by using Las-4000 MINI analyzer (Fuji Photo, Tokyo, Japan) and quantified with Image J software (NIH).

### Behavioral Analysis

As we have described previously (Sohn et al., 2019), memory and learning function of experimental mice was conducted using passive avoidance task (PAT) and Morris water maze (MWM) tests. The PAT was performed using an electronic shock generator with lightened and darkened compartments (Jeungdo Bio and Plant Co. Ltd., Seoul, Korea) on days 8–10 after Aβ injection. Transfer latency time was recorded as the amount of time (within 5 min) the mice remained in the lightened compartment. The MWM was conducted on days 15–21 after Aβ injection. Mice were put in a water pool containing four designated release points and allowed to find the escape platform for 60 s. After finding the platform, the animals were allowed to rest for 30 s on the platform. If mice failed to find the platform within 60 s, they were guided to rest on the platform for 30 s. After all animals finished the first trial, the next trial was started. The release points were randomly chosen every time without overlap. Time taken to find the platform (escape latency) was monitored (training: 2 days, behavioral test: 4 days, and probe trial: 1 day). In the last day (day 7), the platform was removed 1 h after EEFE administration and the probe trial was performed for 60 s to measure the number of times the mice crossed the platform. Experimental mice were given vehicle, EEFE or morin prior to behavioral test.

### Quantitative Analysis of Inflammatory Cytokines in Brain Tissues

Mouse pro-inflammatory cytokines, tumor necrosis factor alpha (TNF-α) and interleukin 1beta (IL-1β) were measured in brain lysates using commercial competitive ELISA kits (Cat. MBS2500421, MyBioSource, San Diego, CA, United States) and (Cat. ab100705, Abcam), respectively. Supernatants of brain lysates were collected by according to manufacturer’s protocol. The concentration of TNF-α and IL-1β was calculated from the standard curves. The optical density (OD) was read using a microplate reader (BioTek Instrument, Winooski, VT, United States) at 450 nm.

### Chemicals, Reagents, and Sample Preparation for Quantitative Analysis

Three standard compounds (rutin, chlorogenic acid, and kaempferol-3-*O*-rutinoside) and Biopurify Phytochemicals were purchased from ChemFaces Biochemical (Wuhan, China) and (Chengdu, China) (purity >98% by high-performance liquid chromatography [HPLC]), respectively. Acetonitrile and water of HPLC grade (J. T. Baker Chemical, Phillipsburg, NJ, United States), and trifluoroacetic acid (TFA, Sigma-Aldrich) were used for quantitative analysis. For quantitative analysis, powdered EEFE leaves were dissolved in 80% aqueous methanol to a final concentration of 10 mg/ml and filtered through a syringe filter (0.45-μm pore size). To make a standard mixture, the stock solutions of the three standard compounds (1.0 mg/ml) were mixed with methanol to final concentration of 0.1 mg/ml and diluted to obtain various concentrations of standard solution before chromatographic analysis.

### Chromatographic Conditions

For HPLC analysis, we used the HPLC system equipped with a photodiode array (PDA) detector (Waters Alliance e2695, PDA #2998, Waters Corp., Milford, MA, United States). The data were acquired and processed using Empower software (version 3; Waters Corp.). For chromatographic separation of the three compounds, a Sunfire C_18_ analytical column (250 × 4.6 mm, 5 μm, Waters Corp.) was used, which was maintained at 35°C. The gradient conditions were 10–23% B for 30 min, 23–100% B for 10 min, and 100% B for 10 min. The mobile phases consisted of two solvents: 0.1% (v/v) TFA in water (A) and acetonitrile (B). For scanning chromatograms, PDA detection was performed at 210–400 nm. Column were used at flow rate of 1.0 ml/min and injected volume of 10 μL, respectively.

### Statistical Analysis

All data were represented as the mean ± SEM. A value of *p* < 0.05 was considered to indicate statistical significance. Prism software (Graph Pad, version 8.4.1, San Diego, CA, United States) was used for all analyses. Statistically significant differences were evaluated with one-way analysis of variance (ANOVA) for comparison three or more groups, and with an unpaired or paired Student’s *t*-test for comparison between two groups. All experiments were performed individually at least three times.

## Results

### Inhibitory Effects of EEFE on Aβ Aggregation

**FIGURE 2:**
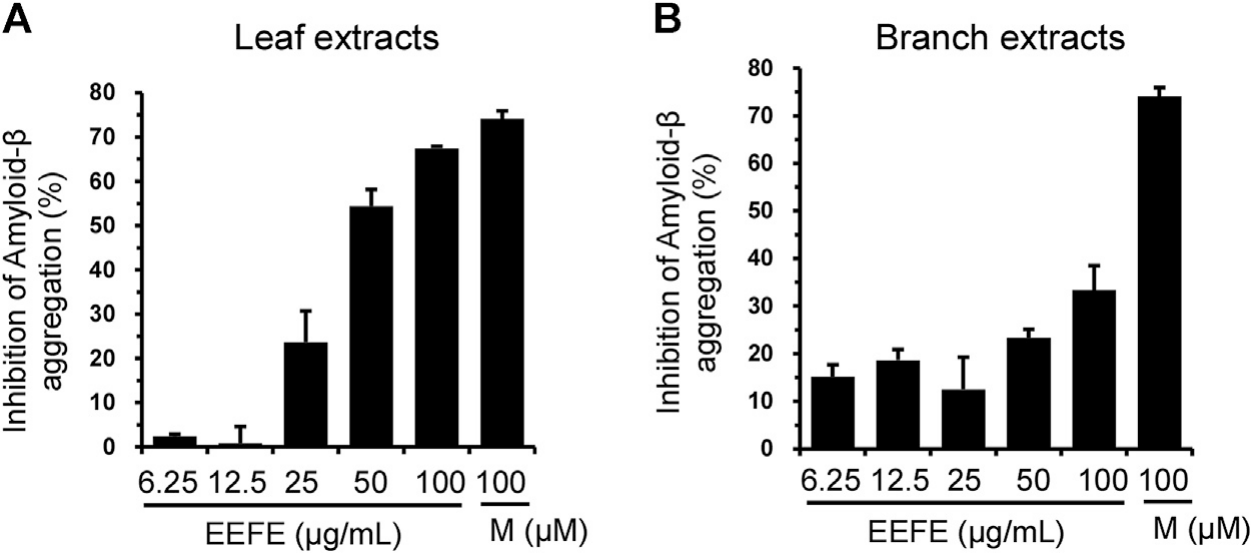
Effects of EEFE leaves and branches on amyloid-β (Aβ) aggregation. **(A, B)** Aβ aggregation assay was performed by reaction with 6.25, 12.5, 25, 50, or 100 μg/ml concentrations of EEFE leaves **(A)** and branches **(B)** with Aβ peptides, followed by the addition of Thioflavin T. Thioflavin T fluorescence was assessed at an excitation 440 nm and an emission 485 nm, and inhibition rate (%) of Aβ aggregation was calculated. Percentage in the Aβ_1–42_ peptide solution without sample was set as 100%. Morin (M) was used as a positive control. EEFE, ethanol extracts of *Ficus erecta*.

Aβ accumulation is a pivotal pathogenic factor in AD progression (Murphy and LeVine, 2010). We assessed the effects of EEFE leaves or branches on Aβ aggregation *in vitro*. In contrast to the branch extracts, the leaf extracts inhibited Aβ aggregation more dramatically. The half maximal inhibitory concentration (IC_50_) values of leaf and branch extracts were 43.43 and >100 μg/ml, respectively (Figures 2A,B).

### Inhibitory Effects of EEFE on Aβ Aggregation in Aβ-Injected Mice

**FIGURE 3:**
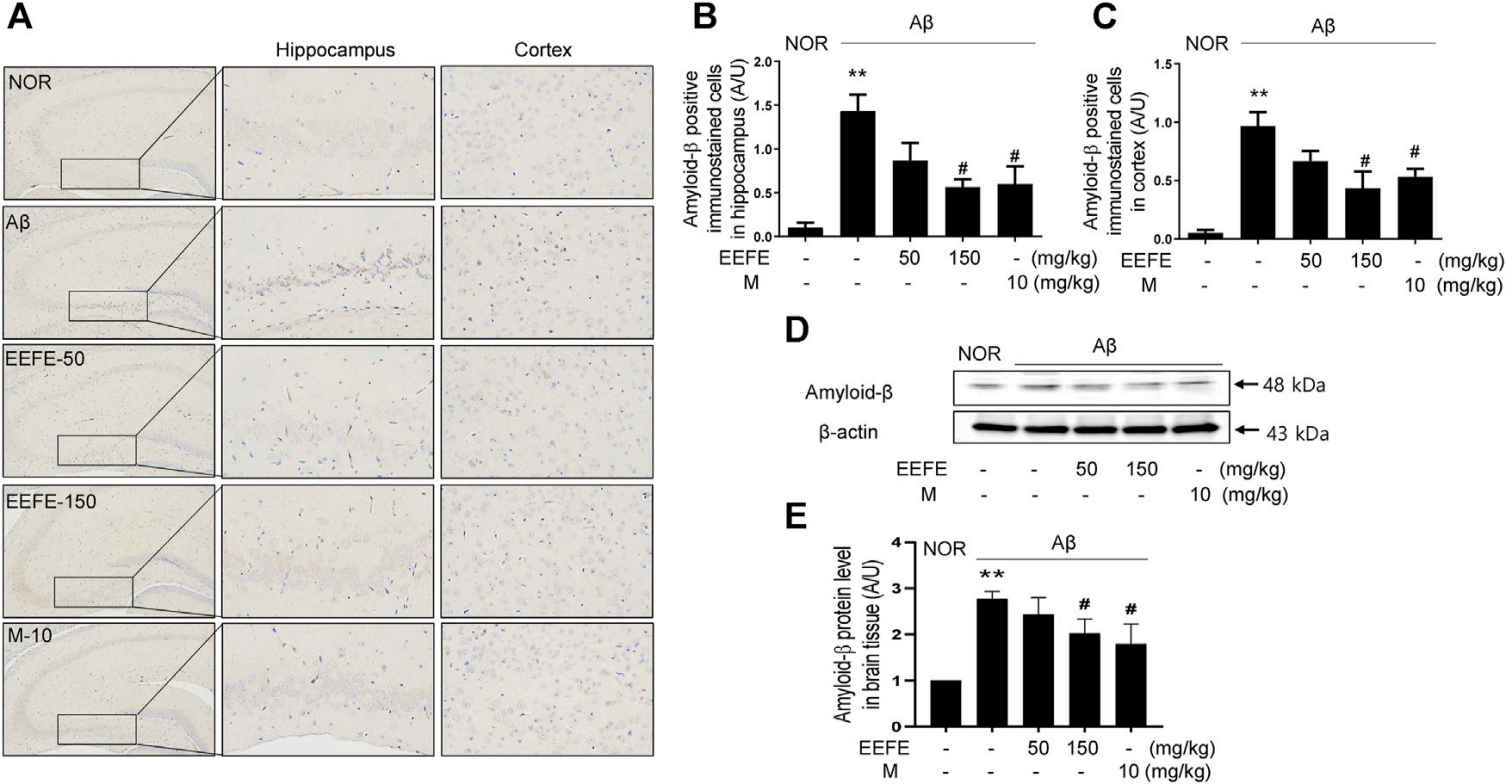
Effects of EEFE on amyloid-β (Aβ) aggregation in Aβ-injected mice. **(A)** Multiple 4-μm paraffin sections of hippocampus and cortex regions were prepared from the sacrificed mouse brains (*n* = 3/group). Aβ expression was determined by immunohistochemistry with Aβ antibody in hippocampal and cortex region of brain sections. Images were captured using a microscope at 400 x magnification (Olympus DP71) **(B, C)**. Quantitative analysis of Aβ positive immunostained cells was performed in hippocampal **(B)** and cortex **(C)** regions of brain sections. Square boxes indicate the magnified pictures of Aβ positive immunostained cells in hippocampal area by brown color DAB stained. The staining intensities are reported in arbitrary unit (AU). **(D)** Western blot analysis was conducted to detect levels of Aβ in brain lysates. **(E)** Quantitative analysis was conducted to assess the band intensities of` Aβ in the western blotting. Data are presented as the mean ± SD (*n* = 4/group). **p < 0.01 vs NOR group and #*p* < 0.05 vs Aβ group. Aβ, Aβ_1–42_–injected mice; EEFE, ethanol extracts of *Ficus erecta*; M, morin; NOR, normal control mice.

The effects of EEFE leaf extracts on Aβ aggregation were further investigated using Aβ-injected mice. Aβ aggregates were delivered through ICV injection into the mouse brain, and EEFE was administered orally for 20 days at 50 or 150 mg/kg/day. Compared with the NOR group, the Aβ group had remarkably more Aβ-positive cells in both hippocampal and cortical regions (Figures 3A–C). One-way ANOVA revealed the significance between group effects (F (4, 10) = 9.39, *p* = 0.002 of hippocampus and F (4, 10) = 11.55, *p* = 0.001 of cortex). In contrast, the EEFE-150 group, but not the EEFE-50 group, had markedly fewer Aβ-positive cells than the Aβ group. Consistently, the EEFE-150 group had significantly decreased expression of Aβ protein in brain tissues compared to the Aβ group (Figures 3D,E). Moreover, one-way ANOVA indicated the significance between group changes in Aβ protein expression of brain tissue (F (4, 15) = 19.44, *p* < 0.001). Morin, positive control, was used for an inhibitor of Aβ aggregation.

### Effects of EEFE on Cognitive Deficit in Aβ-Injected Mice

In our previous study, we performed Y-maze test to verify whether the locomotor activity is affected by Aβ infusion (Lim et al., 2018). According to many studies, Y-maze and open field tests are the most commonly used tests for locomotor activity of rodents by counting the number of entries to the maze arms (Yamada et al., 1999; Zhang et al., 2015; Heredia-López et al., 2016; Postu et al., 2020). Our results showed that Aβ-injected group was not differ from any other groups in total entry number of Y-maze test. These observations suggest that our animal model was not attributed to the differences in their locomotion activities by Aβ infusion (Lim et al., 2018).

**FIGURE 4:**
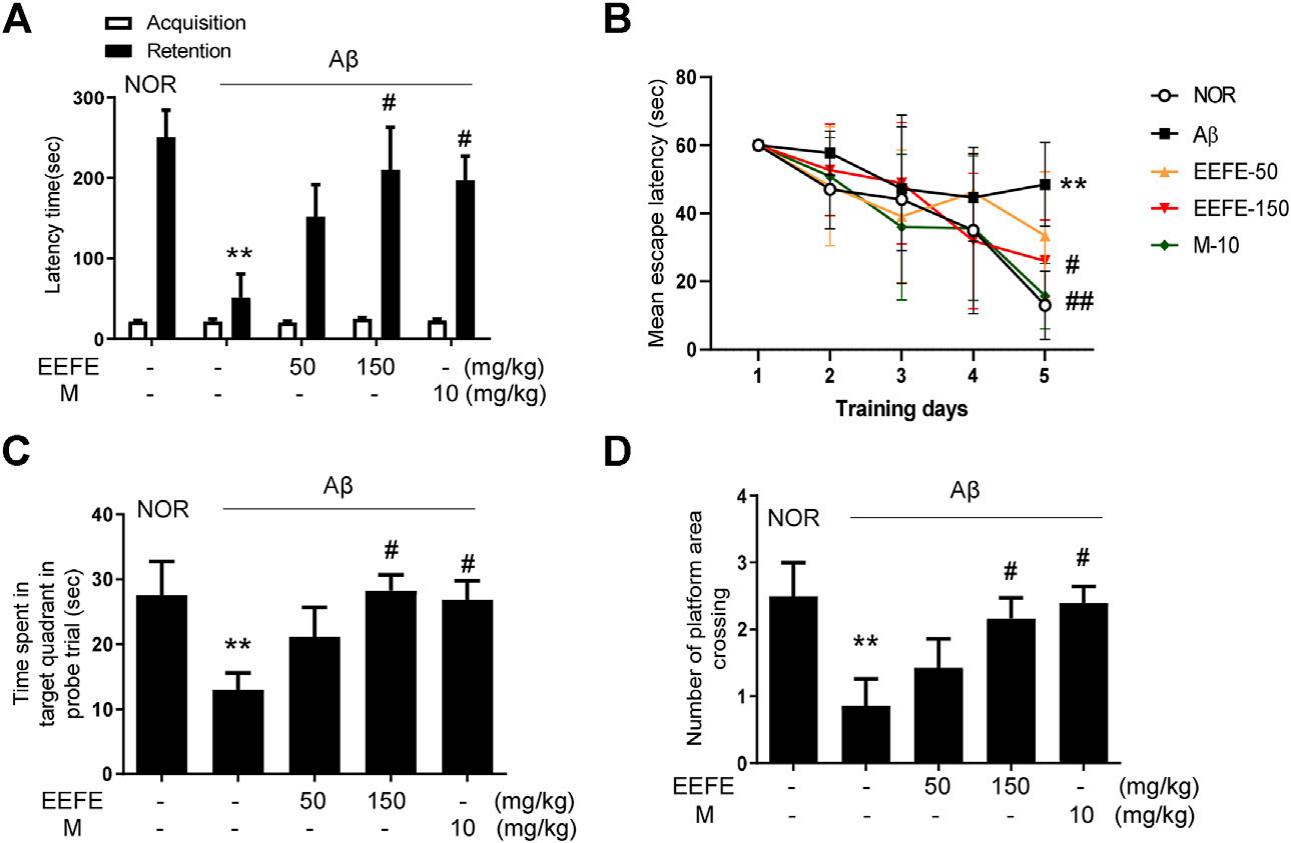
Effects of EEFE on memory and cognitive deficits in Aβ-injected mice. **(A)** The passive avoidance task (PAT) was performed on the 8^th^–10^th^ day after Aβ injection. Transfer latency time was recorded the time mice remain in lighted compartment within 5 min. Morris water maze test (MWM) was conducted on the 15^th^–21^st^ day **(B–D)**. Graphs display the time of spent to find the escape platform during 5 training days **(B)**. The time spent in the target quadrant was measured in the probe trials **(C)**. The number of platform area crossing was evaluated in the probe trials **(D)**. The data are presented as the mean ± SD (*n* = 7/group). **p < 0.01 vs NOR group and ^#^ *p* < 0.05 or ^##^ *p* < 0.01 vs Aβ group. Aβ, Aβ_1–42_–injected mice; EEFE, ethanol extracts of *Ficus erecta*; M, morin; NOR, normal control mice.

To investigate whether EEFE protects against Aβ-induced cognitive deficit in mice, we carried out the PAT and MWM behavioral tests. In the PAT, the transfer latency time was significantly shorter in the Aβ group than in the NOR group. In contrast, the transfer latency time was significantly higher in the EEFE-150 group than in the Aβ group (Figure 4A). One-way ANOVA indicated the significance between group effects (F (4, 25) = 6.545, *p* = 0.001). In the MWM, the mean escape latency time to find the hidden platform was significantly higher in the Aβ group than in the NOR group over the five training days. In contrast, the EEFE-150 group exhibited a marked decline of the latency time compared to the Aβ group (Figure 4B). In the probe trial, the time spent in the target quadrant and platform crossings number were markedly lower in the Aβ group than in the NOR group. In contrast, Aβ-induced negative effects were remarkably reversed in the EEFE-150 group (Figures 4C,D). One-way ANOVA indicated the significance between group effects (Figure 4C; F (4, 25) = 3.159, *p* = 0.03 and Figure 4D; F (4, 25) = 3.631, *p* = 0.0182). These results demonstrate that EEFE improved spatial memory impairment in mice with Aβ-induced cognitive deficit.

### Effects of EEFE on Neuronal Loss in Aβ-Injected Mice

**FIGURE 5:**
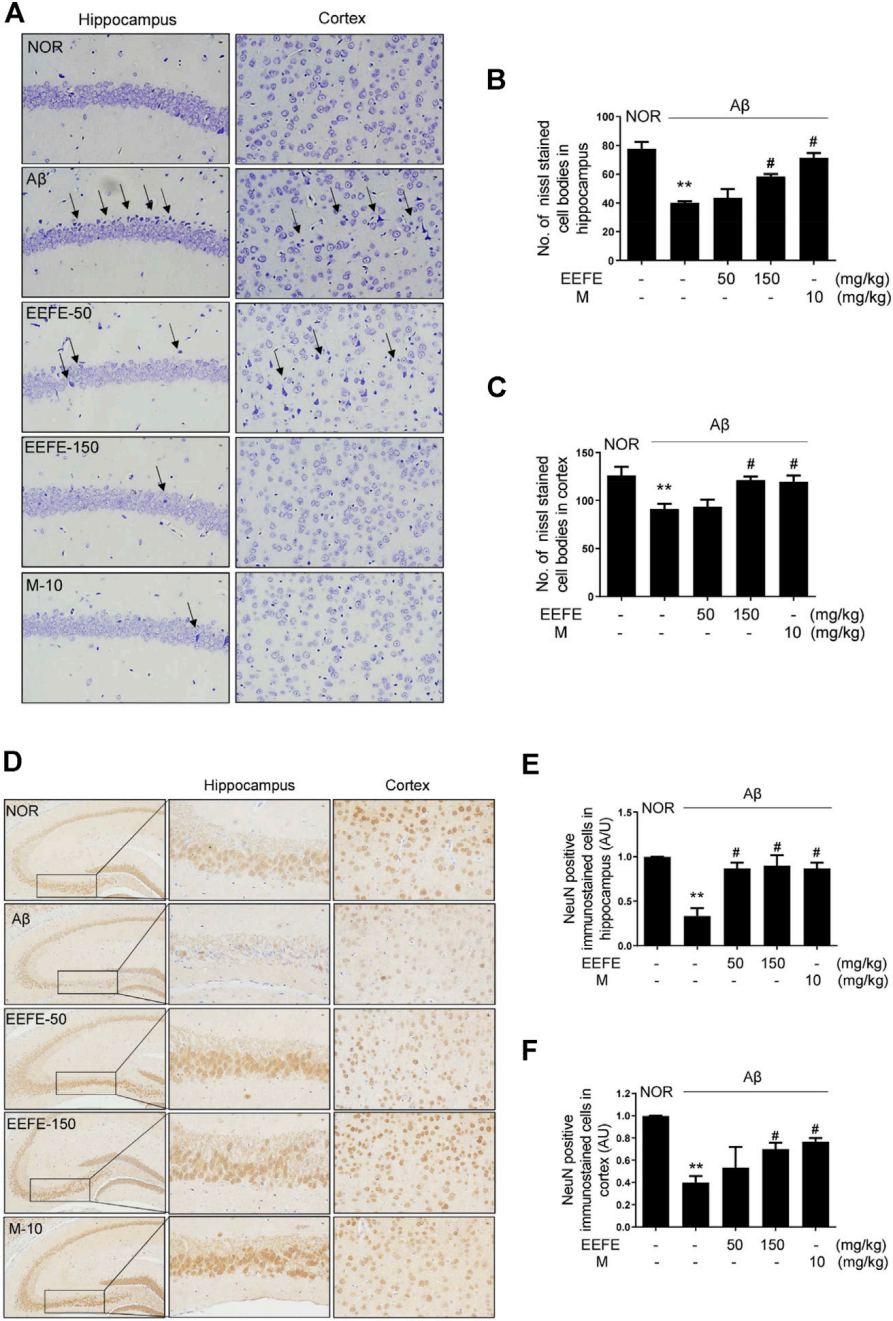
Effects of EEFE on neuronal damage in Aβ-injected mice. Multiple 4-μm paraffin sections of hippocampus and cortex regions were prepared from the sacrificed mouse brains. **(A)** Nissl staining was conducted using cresyl violet solution. **(B, C)** Graphs display the quantitative analysis of Nissl stained cell bodies in hippocampus **(B)** and cortex **(C)**. Black arrows indicate a neuronal shrinkage of Nissl body cells, karyopyknosis. **(D)** The expression of NeuN, as a neuronal marker, was determined by immunohistochemistry. Square boxes indicate the magnified neuronal degeneration in hippocampal area. **(E, F)** Graphs display the quantitative analysis of NeuN positive cells in hippocampus **(E)** and cortex **(F)**. Representative photomicrographs are shown at magnification of ×400. The staining intensities are reported in arbitrary unit (AU). The data are presented as the mean ± SD (*n* = 3/group). **p < 0.01 vs NOR group and #*p* < 0.05 vs Aβ group. Aβ, Aβ_1–42_–injected mice; EEFE, ethanol extracts of *Ficus erecta*; M, morin; NOR: normal control mice.

To determine whether EEFE prevents neuronal loss in mice with Aβ-induced cognitive deficit, we performed Nissl staining and immunohistochemistry for NeuN, a neuronal marker. The number of surviving neurons in the CA1 area of the hippocampus and cortex was markedly reduced in the Aβ group compared to the NOR group, a feature that was accompanied by neuronal shrinkage or loss (Figure 5A). The number of Nissl-stained cell bodies in the CA1 area of the hippocampus (Figure 5B) and cortex (Figure 5C) was significantly higher in the EEFE-150 and M-10 groups than in the Aβ group. One-way ANOVA indicated the significance between group effects (F (4, 10) = 18.79, *p* = 0.001 of hippocampus and F (4, 10) = 6.195, *p* = 0.009 of cortex). Moreover, the number of NeuN-positive cells in the hippocampus and cortex was significantly lower in the Aβ group than in the NOR group (Figures 5D–F). In contrast, neuronal cell loss in the Aβ group was markedly reversed in the EEFE and M groups. One-way ANOVA revealed the significance between group effects Figures 5E,F (NOR, Aβ, EEFE-50, EEFE-150, M-10; F (4, 10) = 11.46, *p* = 0.0009 of hippocampus and F (4, 10) = 6.159, *p* = 0.009 of cortex).

### Effects of EEFE on the CREB/BDNF Pathway in Aβ-Injected Mice

**FIGURE 6:**
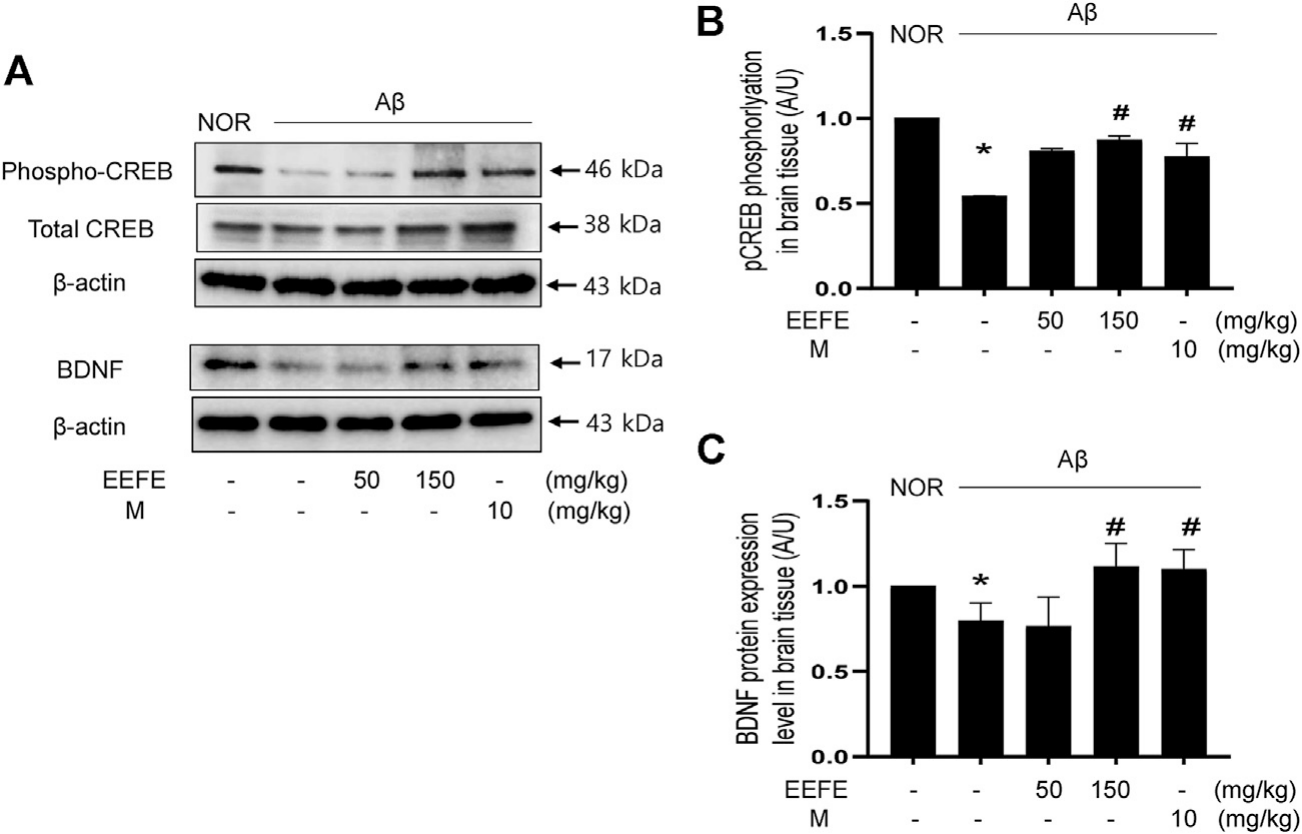
Effects of EEFE on the CREB/BDNF signaling dysfunction in Aβ-injected mice. **(A)** Homogenized brain lysates were applied to western blot analysis. CREB phosphorylation and BDNF expression were detected with anti-phospho-CREB and BDNF antibodies. Images were visualized using a ChemiDox imaging analyzer. **(B, C)** Quantitative analysis was performed to assess the band intensities of phospho-CREB **(B)** and BDNF **(C)** in the western blotting. The protein levels were normalized to β-actin for BDNF and total CREB for phospho-CREB. The results are presented as the mean ± SD (*n* = 3/group). *p < 0.05 vs NOR group and #*p* < 0.05 vs Aβ group. Aβ, Aβ_1–42_–injected mice; EEFE, ethanol extracts of *Ficus erecta*; M, morin; NOR: normal control mice.

The CREB/BDNF pathway is critical in the promotion of neuronal cell survival related with memory and learning ability in AD (Platenik et al., 2014). We determined the expression of BDNF and phosphorylation of CREB by western blot analysis. One-way ANOVA revealed the significance between group effects (F (4, 10) = 57.96, *p* < 0.001) in phospho-CREB and (F (4, 10) = 5.564, *p* = 0.012) in BDNF expression. In the Aβ group, the levels of phospho-CREB and BDNF in brain tissue lysates were markedly reduced compared to those in the NOR group. However, Aβ-induced suppression of CREB phosphorylation and BDNF expression was significantly reverted in the EEFE-150 group (Figures 6A–C). Positive control morin also had inhibitory effects on the CREB/BDNF signaling pathway.

### Effects of EEFE on Inflammatory Protein Expression in Aβ-Injected Mice

**FIGURE 7:**
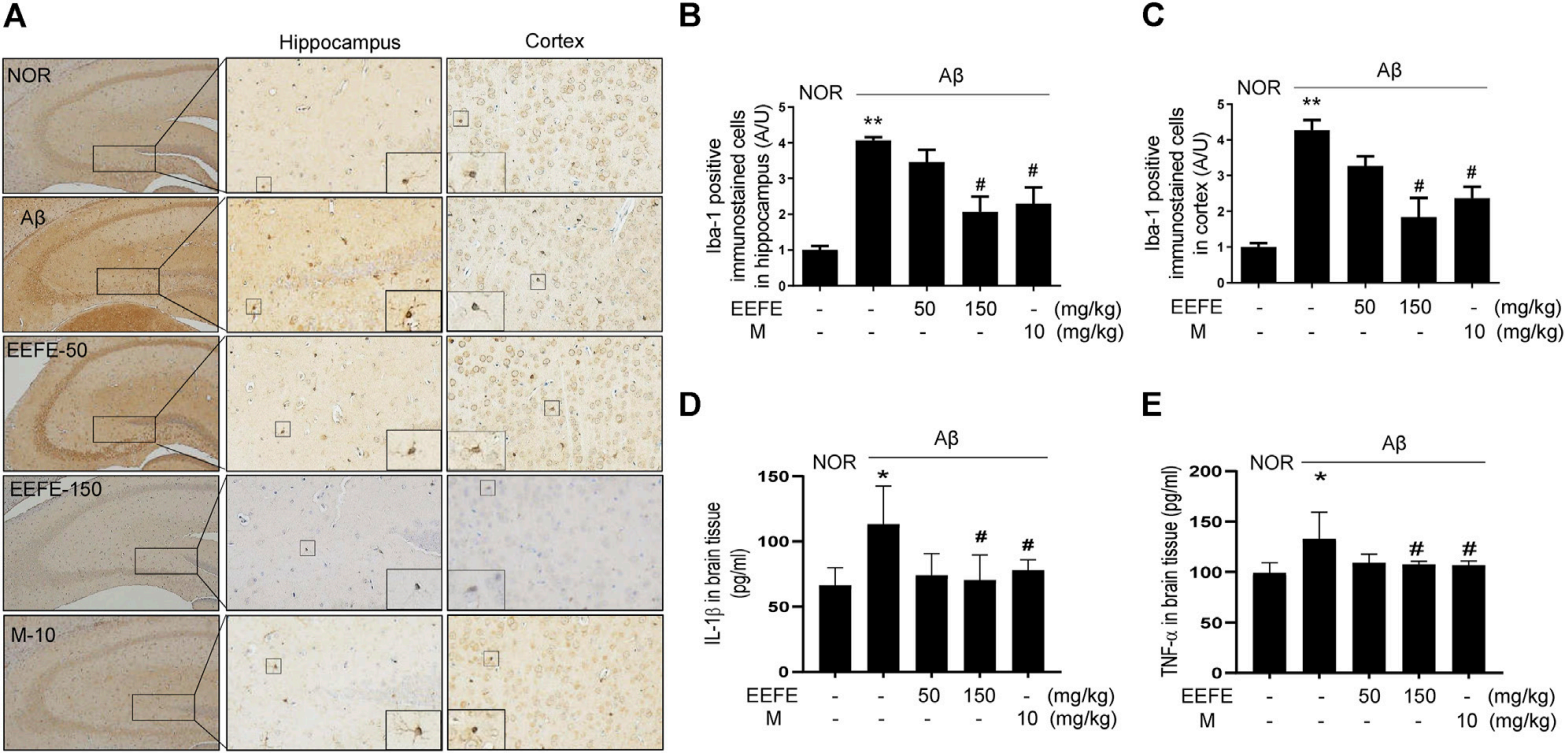
Effects of EEFE on the neuroinflammation in Aβ-injected mice. **(A)** Multiple 4-μm paraffin sections of hippocampus and cortex regions were prepared from the sacrificed mouse brains (*n* = 3/group). Expression of Iba-1, known as marker of microglical activation, was determined by immunohistochemistry with Iba-1 antibody in hippocampal and cortex region of brain sections. **(A)** Images were captured using a microscope at 400 x magnification (Olympus DP71). **(B, C)** Quantitative analysis of Iba-1 positive immunostained cells was performed in hippocampal **(B)** and cortex **(C)** regions of brain sections. Square boxes indicate the magnified pictures of Iba-1 positive cells. The staining intensities are reported in arbitrary unit (AU). The data are presented as the mean ± SD (*n* = 3/group). **(D, E)** Levels of inflammatory cytokines IL-1β (*n* = 4/group) and TNF-α (*n* = 4/group) in brain lysates were determined using ELISA kits according to the manufacturers’ instructions. The data of three independent experiments are expressed as mean ± SEM. *p < 0.05 or **p < 0.01 vs NOR group and #*p* < 0.05 vs Aβ group. Aβ, Aβ_1–42_–injected mice; EEFE, ethanol extracts of *Ficus erecta*; M, morin; NOR: normal control mice.

Neuroinflammation plays an essential role in the pathogenesis of AD (Heneka et al., 2015). We investigated whether EEFE protected against neuroinflammation in Aβ-injected mice. Immunohistochemistry and ELISA were carried out to assess the levels of Iba-1, known as maker of microglia cell activation, and pro-inflammatory cytokines IL-1β and TNF-α in the brain, respectively. Iba-1 positive cells were markedly enriched in the hippocampus and cortex region of the Aβ group compared with the NOR group (Figures 7A–C). One-way ANOVA revealed the significance between group effects (F (4, 10) = 14.12, *p* = 0.0004 in hippocampus and F (4, 10) = 14.03, *p* = 0.0004). Consistently, the levels of IL-1β and TNF-α in the brain lysates were significantly elevated in the Aβ group compared with the NOR group. Regarding the effect of EEFE on the inflammation of Aβ induced brain, one-way ANOVA indicated the significance between group effects (F (4, 15) = 4.237, *p* = 0.017 in IL-1β and F (4, 15) = 5.884, *p* = 0.004 in TNF-α). In contrast, the EEFE-150 group significantly reversed the levels of Iba-1, IL-1β, and TNF-α compared to the Aβ group (Figure 7). Morin showed anti-neuroinflammatory effects.

### Determination of the Three Standard Compounds in EEFE

**FIGURE 8:**
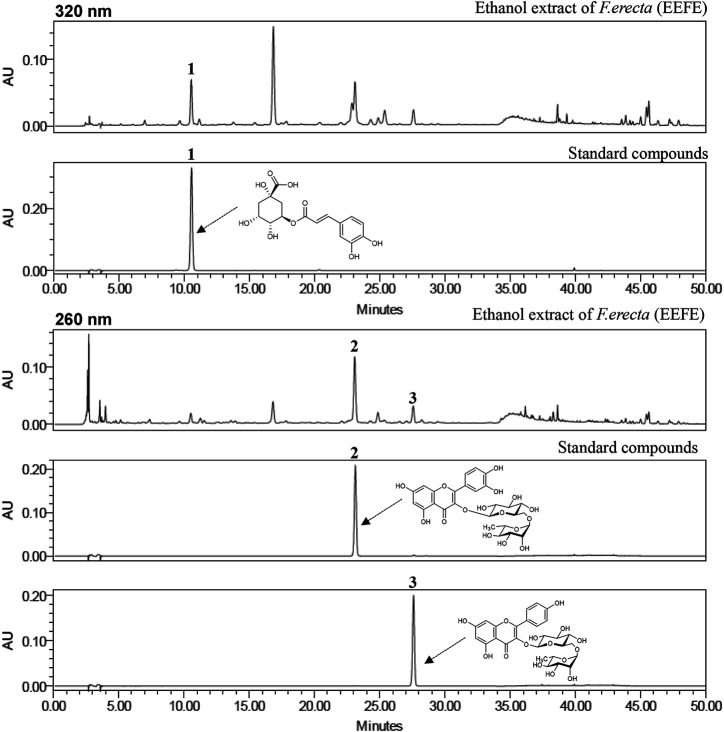
HPLC chromatograms of EEFE and standard mixture at 260 and 320 nm chlorogenic acid (1), rutin (2), and keampferol-3-O-rutinoside (3).

The simultaneous determination of the three standard compounds in EEFE was performed by an optimized HPLC method. The chromatograms showed good separation using mobile phases consisting of 0.1% (v/v) TFA in water and acetonitrile. The ultraviolet (UV) wavelengths to detect compounds were 320 nm for chlorogenic acid, and 260 nm for rutin and kaempferol-3-*O*-rutinoside. The retention times of chlorogenic acid, rutin, and kaempferol-3-*O*-rutinoside were 10.71, 23.48, and 27.97 min, respectively. The three compounds were resolved within 30 min. The HPLC chromatograms of EEFE and the standard mixture are presented in Figure 8. The calibration curves for the three compounds were calculated by the linear relationships between the concentration (x, μg/ml) ranging from 3.125–100 μg/ml and peak area (y) for each standard compound and presented as regression equations (y = ax + b) in Table 1. All correlation coefficient values showed good linearity (r^2^ ≥ 0.9999). The limit of detection (LOD) for the three compounds ranged from 0.185–0.526 μg/ml and the limit of quantitation (LOQ) ranged from 0.562–1.595 μg/ml. The amounts of chlorogenic acid, rutin, and kaempferol-3-*O*-rutinoside in EEFE were 1.80, 5.49, and 1.38 mg/g, respectively (Table 1).

**TABLE 1:** Regression equation, linearity, LOD, LOQ, and content of three compounds.

Compound	Linear range (μg/ml)	Regression equation (*y* = a*x* + B)^a^		r^2^	LOD^b^ (μg/ml)	LOQ^c^ (μg/ml)	Content (mg/g)
		Slope (a)	Intercept (B)				
Chlorogenic acid	3.125–100	33760	−85.066	0.9999	0.185	0.562	1.80 ± 0.01
Rutin	3.125–100	21010	8588.4	0.9999	0.443	1.343	5.49 ± 0.02
Kaempferol-3-O-rutinoside	3.125–100	21232	8269.3	0.9999	0.526	1.595	1.38 ± 0.01

^a^ *y* = a*x* + b, *y* means peak area and *x* means concentration (μg/ml).

^b^ LOD: 3. × (standard deviation (SD) of the response/slope of the calibration curve).

^c^ LOQ: 10 × (SD of the response/slope of the calibration curve).

## Discussion

In current study, we explored for the first time the pharmacological effects of *F. erecta* leaves in AD or AD-related diseases. We found that EEFE prevented Aβ aggregation, improved memory and cognitive dysfunction, and had neuroprotective and neuroinflammatory effects in a mouse model of Aβ-induced AD.

Aβ, a key molecule in the AD pathogenesis, leads to neurotoxicity and neuronal loss (Park et al., 2018), and increases production of Iba-1, TNF-α, and IL-1β levels, known as inflammatory factors, in the brain of patients with AD (Akiyama et al., 2000). The main symptom of AD is memory and cognitive deficit (Choi et al., 2012; Morroni et al., 2018). Aβ-induced AD mouse is a well-characterized AD-like model that is characterized by learning and memory impairment as well as neuroinflammation and neuronal damage (Wang et al., 2017; Du et al., 2018), supporting its validity as a model for AD (Kim et al., 2018). Accumulating behavioral data demonstrate that direct injection of neurotoxic Aβ aggregates into the mouse brain causes memory and cognitive deficit (Choi et al., 2018; Lim et al., 2019). On the basis of these results, we conducted PAT and MWM behavioral tests in mice with Aβ-induced AD treated with or without EEFE for 20 days. Consistent with previous reports (Lim et al., 2019), our results demonstrate that Aβ injection significantly enhanced memory and cognitive deficits in the MWM and PAT whereas EEFE treatment markedly reversed Aβ-mediated cognitive deficit, suggesting that EEFE could protect against memory and cognitive deficits in AD-like mice.

Memory and cognitive deficits are associated with the interplay between hippocampal and cortical regions caused by Aβ accumulation (Preston and Eichenbaum, 2013; Wilson et al., 2017). Death of neuronal cells by Aβ accumulation in the prefrontal brain area has been regarded as an early event in patients with AD (Trujillo-Estrada et al., 2014; Tonnies and Trushina, 2017). That is, hippocampal and cortical damage might contribute to memory and cognitive deficits in AD. In the present study, we found that EEFE inhibited Aβ aggregation in the brain of Aβ-injected mice. Accumulation of Aβ aggregates in the mouse brain was visualized using immunohistochemistry and western blotting. EEFE treatment significantly reduced the number of Aβ-positive cells in hippocampal and cortical regions, and decreased the level of Aβ protein in brain lysates of Aβ-injected mice. The inhibitory effects of EEFE on Aβ aggregation were further confirmed in an *in vitro* Aβ aggregation assay using thioflavin T. Moreover, Nissl staining and NeuN immunohistochemistry revealed neuronal damage and loss in hippocampal and cortex regions exposed to Aβ ICV injection. However, EEFE treatment significantly reversed neuronal/NeuN-positive cell loss in brain tissues. These results indicate the protective effects of EEFE against Aβ-induced neurotoxicity in an AD mouse model.

The CREB/BDNF signaling pathway is one of common neuroprotective mechanism in AD. Changes in CREB level are associated with the pathophysiology of various neurodegenerative diseases (Platenik et al., 2014; Ma et al., 2019). CREB phosphorylation is responsible for transcriptional activation, leading to the production of BDNF, which in turn plays key roles in cognitive functions and synaptic plasticity involved in short- and long-term memory formation. Thus, reducing BDNF/CREB levels is thought to be an important component of AD pathology mediated by neurotoxic Aβ production (Hubka, 2006; Scott Bitner, 2012; Giuffrida et al., 2018). In this study, we found that EEFE treatment reverted the reduction in phospho-CREB and BDNF protein levels in brain lysates of AD-like mice. These results suggest that EEFE-mediated memory and cognitive impairment may be associated with activation of the CREB/BDNF signaling. Actually, many studies have reported that CREB/BDNF activation in the brain of experimental mice facilitates successful memory retrieval (Weon et al., 2016; Wang et al., 2018; Ma et al., 2019), implying that enhancers of CREB/BDNF pathway may have the potential for treatment of AD. In this respect, EEFE may be considered one of these candidate drugs. Additional experiments will be necessary to verify the precise molecular mechanisms of EEFE to inhibit memory and cognitive impairment in our next report.

Neuronal inflammation plays an essential role in facilitating Aβ deposition, neuronal loss, and cognitive deficit (Calsolaro and Edison, 2016; McGrattan et al., 2019; Nordengen et al., 2019). Many studies suggested that inhibition of pro-inflammatory cytokines may be a valuable strategy for subverting AD progression in preclinical and clinical trials (Wang et al., 2015). In addition, recent studies have reported that inflammatory cytokines TNF-α, IL-1β, and IL-6 are produced by activated microglia, which are linked to Aβ-mediated AD pathology. Pro-inflammatory cytokines are pivotal contributors to early stage AD pathogenesis; they can lead to neuronal injury and cell death (Calsolaro and Edison, 2016; Boza-Serrano et al., 2018). In this regard, upregulation of neuroinflammatory factors is considered a pathological factor of AD that may be targeted by drugs to overcome cognitive deficits. Our results showed that the levels of Iba-1, an activation marker of microglia, and pro-inflammatory cytokines TNF-α and IL-1β, were increased in Aβ-injected brain tissues. EEFE treatment markedly reduced the number of Iba-1-positive cells in hippocampal and cortical regions, and the level of inflammatory cytokine in brain lysates of AD-like mice. These results demonstrate that the beneficial effects of EEFE against neuroinflammation might reduce neuronal injury in AD-like mice.

EEFE was effective at 150 mg/kg, but not at 50 mg/kg. However, EEFE was not associated with toxic side effects during the experimental period—not even at 150 mg/kg. The activity of EEFE-150 was comparable to that of morin, a positive control of Aβ inhibitors. Crude plant extracts or medicinal plants with various flavonoids and alkaloids compounds have been shown to have neuroprotective effects against AD or AD-like conditions (Habtemariam, 2019), and *F. erecta* is one such medicinal plant (Yoon et al., 2007; Park et al., 2012; Yakushiji et al., 2012; Abullah et al., 2018). Our study firstly reports the advantageous effects of EEFE on memory improvement and neuroprotection in AD-like mice. By using HPLC analysis, we identified rutin, chlorogenic acid, and keampferol-3-*O*-rutinoside (nicotiflorin) as standard compounds of EEFE. The most abundant compound in EEFE was rutin (5.49 ± 0.02 mg/g), which of the three standard compounds in EEFE by identified using the quantitative analysis of HPLC–PDA method. We and others have reported that rutin has strong neuroprotective effects due to anti-oxidant, anti-amyloidogenic activities, and anti-inflammatory activities in neurodegenerative diseases, including AD (Habtemariam, 2016; Lim et al., 2019). Keampferol-3-*O*-rutinoside also attenuates memory dysfunction via neuroprotective effects against oxidative stress and inflammation (Li et al., 2006; Huang et al., 2007). Chlorogenic acid has the potential to protect against neurological degeneration involved with oxidative stress and neurotoxicity in the brain (Mikami and Yamazawa, 2015; Heitman and Ingram, 2017). Blood-brain-barrier (BBB) permeabilization considered one of the important step in the treatment of related to neurodegenerative diseases (Daneman and Prat, 2015; Bagchi et al., 2019). In present study demonstrate that molecular weights of the three major compounds keampferol-3-o-rutinoside (MW: 594.52), chlolorogenic acid (MW: 354.31), and rutin (MW: 610.52), respectively. Recently, Keampferol-3-O-rutinoside (Ma et al., 2017) and cholorogenic acid (Nabavi et al., 2017) has a good ability to cross the BBB in brain cell *in vitro* or *in vivo*. Rutin and/or its metabolites has abilities to cross the BBB (Habtemariam, 2016) in AD model and has preventative effect of BBB disruption in animal model (Jang et al., 2014). Based on these findings, effects of EEFE against neurodegeneration might be thought as a result of the synergistic interaction of the constituent components (chlorogenic acid, rutin, and keampferol-*O*-3-rutinoside). Further study will be needed to find more compounds in unidentified peaks of HPLC chromatograms and determine the potential bioactive compound(s) of EEFE.

In summary, EEFE protects against cognitive deficit and neuronal damage in mice with Aβ-induced neurotoxicity and inflammation. The pharmacological effects of EEFE occurs via activation of the CREB/BDNF signaling pathway and upregulation of inflammatory cytokines. Overall, our findings support that EEFE is a promising drug candidate for the treatment or prevention of AD or AD-related neurodegenerative conditions.

## Data Availability

The original contributions presented in the study are included in the article, further inquiries can be directed to the corresponding authors.
